# Supplementary material for: Extreme value theory as a framework for understanding mutation frequency distribution in cancer genomes
Source: PLoS One. 2021 Aug 23;16(8):e0243595. doi: 10.1371/journal.pone.0243595 (PMC8382180; doi:10.1371/journal.pone.0243595)
Supplement: S1 Fig — (PDF) [file pone.0243595.s001.pdf]

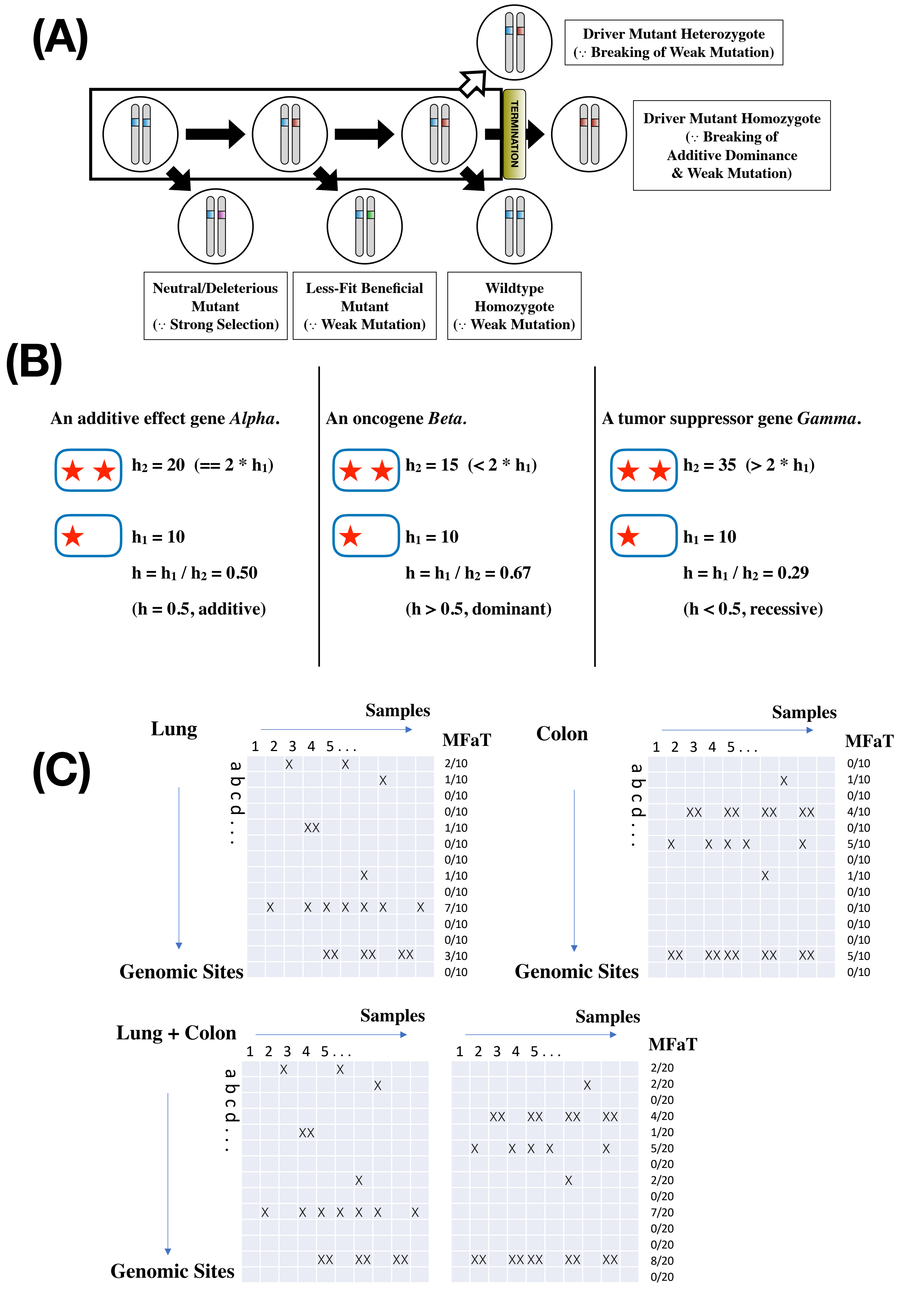

S1 Fig. The SSWM population dynamics of cells and the additivity of fitness effects of mutant alleles.

(A) The SSWM dynamics of preclonal cancer cells. In the SSWM dynamics, we focus on only cells with the maximum fitness. Neutral and deleterious mutants are excluded from consideration due to *strong selection* (the cell with a *purple* mutation). Similarly, a less-fit beneficial mutant (the cell with a *green* mutation) is also excluded because we assume sequential fixation of mutations in a small and non-fluctuating population (i.e., *weak mutation*). In preclonal cancer cells, there are possibly chromosomal missegregation of the diploid cells. This may produce a wildtype homozygote that will be similarly excluded as well and a driver mutant homozygote (the cell with two *red* mutations) that may drive tumorigenesis. In the loss-of-heterozygosity (LoH) model, chromosomal deletion is also possible. A driver event may confer a great fitness gain to the cell to cause a rapid population increase, breaking the *weak mutation* limit. The large empty box shows the scope of the SSWM dynamics. Circles indicate cells. The vertical bars indicate a copy of the cell's genome. Colored boxes on the bars indicate mutations.

(B) Additivity and non-additivity of fitness effects of mutant alleles. Example cases for imaginary genes (i.e., *Alpha*, *Beta*, and *Gamma*) are shown. In the MSB model, the degree of dominance and recessiveness is quantified as dominance coefficient. Fitness effects of the two mutant alleles at the same genomic locus are either additive or non-additive. If such effects are additive, the fitness effect of a heterozygous locus is exactly an intermediate of the two associated homozygotes. In this case (i.e., the gene *Alpha*), the dominance coefficient equals 0.5. Activating mutations in a typical oncogene (i.e., the gene *Beta*) are believed to be dominant in relation to the cell's malignant phenotype. In this case, the fitness effect of a homozygous mutant locus is smaller than the sum of the two heterozygotes. Thus, the dominance coefficient is greater than 0.5 and the mutant allele is considered dominant. In contrast, loss-of-function mutations in a typical tumor suppressor gene (i.e., the gene *Gamma*) is believed to be recessive. In a heterozygous mutant, the cell has one copy of genome with a non-functional mutant, and one remaining functional. In a homozygous mutant, on the other hand, the cell has both copies mutated, with a greater fitness effect than the sum of the two heterozygotes. In this case, the dominance coefficient is smaller than 0.5 and thus showing recessiveness. This phenomenon has been extensively studied as Knudoson's Two-Hit hypothesis, LoH, and other related concepts. Stars indicate mutations. Circles indicate cells.

(C) A schematic of our calculation method. From our assumptions, the frequency of each value of MFaTs will follow the extreme value distribution. The analysis of the actual data revealed that the distribution seems to be indeed the Fréchet-type of the extreme value distribution. In the figure, *X* indicates a mutation in one allele.
