# Supplementary material for: Extreme value theory as a framework for understanding mutation frequency distribution in cancer genomes
Source: PLoS One. 2021 Aug 23;16(8):e0243595. doi: 10.1371/journal.pone.0243595 (PMC8382180; doi:10.1371/journal.pone.0243595)
Supplement: S2 Fig — (PDF) [file pone.0243595.s002.pdf]

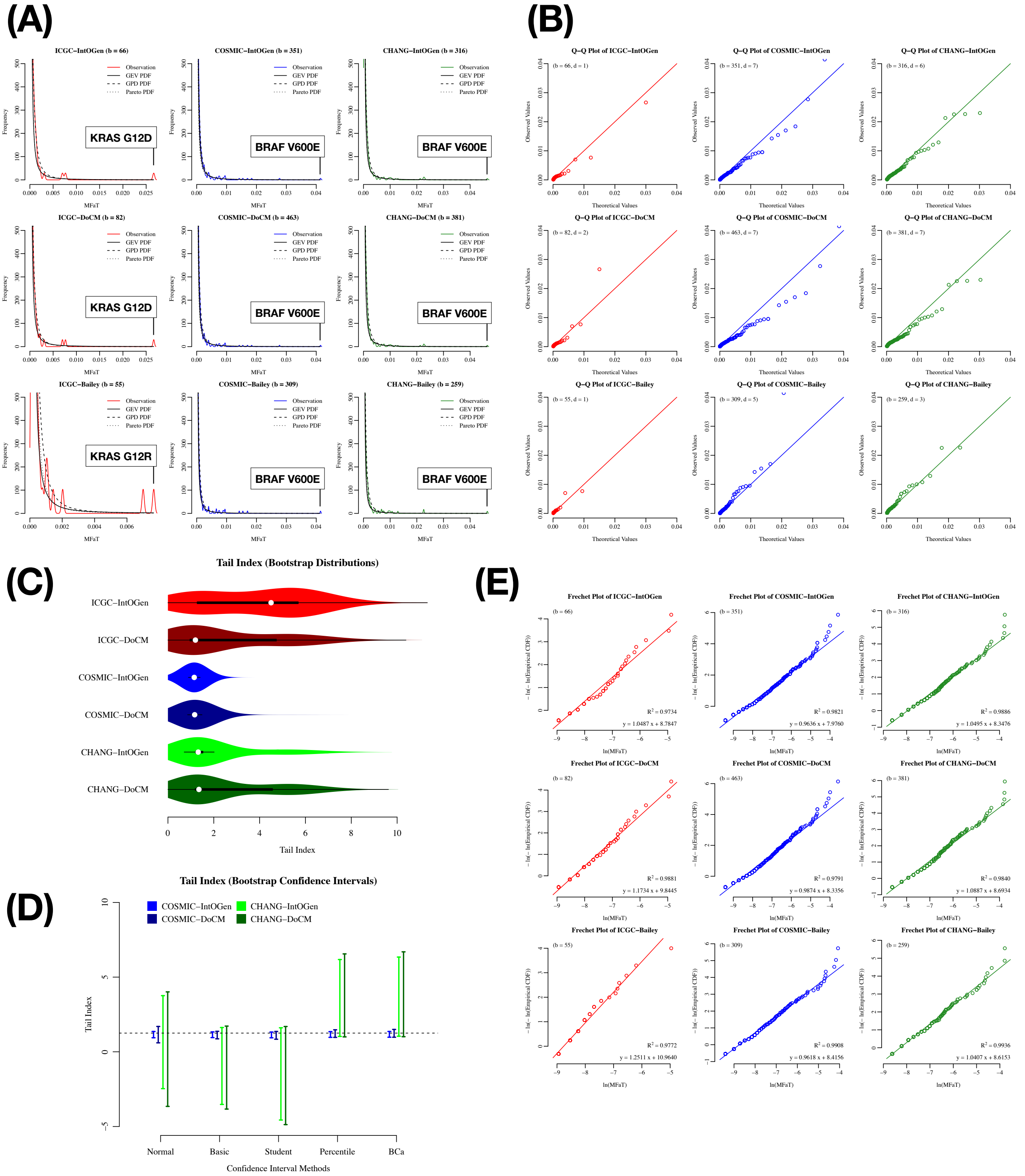


**S2 Fig. Exploratory plots on cancer driver mutation MFaTs in the total-tumor analysis with position-based filtering.**

In these figures, symbols such as ``ICGC-DoCM'' are similar to Fig 2. However, the symbol ``IntOgen,'' for example, denotes a driver-site definition (i.e., position-based filtering)(for details, see Materials and Methods).

(A) Density plot of MFaTs. The colored solid line is the probability density of observations, the black solid line is the probability density function (PDF) of GEV, and the dotted lines are the PDFs of the GPD and Pareto distributions. For each plot, a gene symbol and an amino acid substitution of a mutation with the highest MFaT value is shown in a box. Disagreement between the observed and theoretical densities in the case of ICGC-Bailey is likely due to the lack of data points ($b$ = 55).

(B) Q-Q plot of MFaTs. Here, ``b'' denotes the number of genomic sites of beneficial mutations considered, and ``d'' denotes the parameter for normalization in the Q-Q plot. The straight line has the equation $y = x$. In the Q-Q plot, each pair of an observation and a theoretical value corresponds to the respective points.

(C) Bootstrap distributions of the tail index. The white point indicates the median of the distribution. The black square shows the first and the third quantiles. We excluded the cases of the Bailey position-based filtering from the process due to their lack of data points.

(D) Bootstrap confidence intervals. The dotted line shows the value that is in range of all confidence interval cases. Cases with the ICGC mutation frequency data and Bailey position-based filtering were omitted from the calculation.

(E) Fréchet plot. The $R^2$ values and the equation for the linear regression lines are shown. The symbol ``b'' is defined as in (B).
