# Supplementary material for: Extreme value theory as a framework for understanding mutation frequency distribution in cancer genomes
Source: PLoS One. 2021 Aug 23;16(8):e0243595. doi: 10.1371/journal.pone.0243595 (PMC8382180; doi:10.1371/journal.pone.0243595)
Supplement: S1 Appendix — (PDF) [file pone.0243595.s003.pdf]

S1 Appendix

Supplementary Materials

Data Processing in Detail

In the case of the ICGC dataset, we used the Release 27 Summary Simple Somatic Mutation VCF file, for which the reference genome build is GRCh37 and mutation annotations are based on Ensembl version 75. First, we selected protein-altering mutations (annotated as either "missense_variant," "stop_gained," "stop_lost," or "initiator_codon_variant") in the VCF file using our custom script (see S1 Script: get_IcgcProteinAltering.py). If a mutation had multiple annotated transcripts, and it had been annotated as a protein-altering mutation in any of those transcripts, we included such a mutation in the analysis because it will satisfy the *strong selection assumption* in cancer driver genes. Next, we selected doubleton (affected_donors > 1) SNV records and then calculated and maximized their MFaTs (donor-based MFaTs). For the ease of the process, we added gene symbols as an independent column that had been a part of the VCF annotations (based on Ensembl 75)(see S1 File: database_ICGC_temp_PostMax.tsv.gz). The fields of the affected donor count (affected_donors) and the total donor count (total_donors, 15 285) are used as the mutated tumor count and the total tumor count in an MFaT calculation, respectively.

In the case of the COSMIC dataset, we used the GRCh37 Version 85 Mutant Export TSV file that includes mutations from cancer cell lines. First, we selected SNVs that were doubletons or more frequent (CNT > 1) from the coding mutation VCF (with redundancy due to annotations) based on mutation IDs. Among those mutant export TSV records that had mutation IDs, we analyzed only those obtained from genome sequencing data ("Genome-wide screen" == "n") mapped to the GRCh37 genome build (GRCh == "37"). We summed mutation IDs based on tumor IDs and calculated the MFaTs for the respective mutations (tumor-based MFaTs). For these mutation records, we added genomic coordinates using the VCF file, formatted the records appropriately, and then removed redundancies due to annotated gene symbols. Finally, we selected only protein-altering mutations (in practice, those with recorded amino acid substitutions) and maximized the calculated MFaTs (see S2 File: database_COSMIC_temp_PostMax.tsv.gz). The fields of the affected tumor count (affected_tumors) and the total tumor count (total_tumors, 24 355) were used as the mutated tumor count and the total tumor count in an MFaT calculation, respectively.

In the case of the CHANG dataset, we used the PanCancer Unfiltered MAF file. Out of all mutations recorded in the file, we selected only records with mutations that were protein-altering (annotated as either "missense_variant," "initiator_codon_variant," "stop_gained," or "stop_lost") SNVs and potential doubletons according to summed sample IDs. We added total sample counts for those records based on sample IDs, calculated MFaTs totaling sample IDs for each mutation (sample-based MFaTs), and then maximized these MFaTs (see S3 File: database_CHANG_temp_PostMax.tsv.gz). The fields of the affected sample count (affected_samples) and the total sample count (total_samples, 11 089) were used as the mutated tumor count and the total tumor count in the MFaT calculation, respectively.

We selected driver mutations according to gene symbols in the case of the driver-gene definitions and to genomic coordinates in the case of driver-site definitions. We generated driver-gene definition files for the IntOgen, CGC, and Tokheim datasets,

respectively (for details, see Datasets section and S4 File: driver_TotalGene_database_IntOGen.tsv, S5 File: driver_TotalGene_database_CGC.tsv, S6 File: driver_TotalGene_database_Tokheim.tsv). Also, we generated driver-site definition files for the IntOgen, DoCM, and Bailey datasets, respectively (for details, see Datasets section and S7 File: driver_TotalSite_database_IntOGen.tsv, S8 File: driver_TotalSite_database_DoCM.tsv, S9 File: driver_TotalSite_database_Bailey.tsv).

In the case of the RTCGA mutations dataset, we used a reformatted file by selecting the necessary columns after processing the header row (i.e., we renamed a column from "Start_position" to "Start_Position") (see S10 File: database_RTCGA_temp_Format.tsv.gz).

Further, we select protein-altering SNV records whose reference gnome build is GRCh37 (NCBI_Build == "37"), sequencing strategy is whole exome sequencing (Sequence_Source == "WXS"), VAF (Variant Allele Frequency) is greater than 0.25 (VAF > 0.25), and mutation class is either "Missense_Mutation," "Nonsense_Mutation," "Translation_Start_Site," or "Nonstop_Mutation" (see S11 File: database_RTCGA_temp_ProteinAlteringSnv.tsv.gz) (for details of the RTCGA dataset at this step, see Datasets section). Finally, we calculated total sample counts (total_samples) per tumor type in a reference/alternate-sensitive manner and selected mutation records that are potential doubletons (see S12 File: database_RTCGA_temp_ProteinAlteringSnvDoubleton.tsv.gz). In the calculation of MFaTs, the total sample counts (total_samples) and the affected sample counts (affected_samples) are used as total tumor counts and mutated tumor counts.

In the case of RTCGA total-tumor analysis, we summed sample IDs over all tumor types (affected_samples) across the RTCGA dataset, calculated MFaTs, and subsequently maximized them (see S13 File: database_RTCGA_temp_PostMaxTotal.tsv). The method of extracting the intersection set of mutations about the driver-gene and driver-site definitions is similar to the cases of ICGC and other datasets.

In the case of RTCGA type-specific analysis, we summed sample IDs, calculated MFaTs, and maximized them in a tumor-type-specific manner (affected_samples)(see S14 File: database_RTCGA_temp_PostMaxType.tsv). We used only driver-gene definitions in extracting the intersection set of mutations (see S15 File: driver_TypeGene_database_IntOGen.tsv). In this way, we initially generated a list of genes within the driver-gene definition, given a certain tumor type, and then selected mutations that occurred within the listed genes (see S16 File: drivertype_RTCGA-IntOGen_temp_PreRank.tsv).

Cancer Driver MFaT Is the Expectation of Driver Mutant VAFs

The relationship of MFaT with VAF is as follows. When a large-scale genome dataset is given, we write a VAF of a certain genomic site f and the conditional expectation of VAF g under a condition that the genomic site is specified. Here we call this g "aggregated tumor VAF" or ATVAF. In the below formula, T denotes the set of tumor samples considered, M is the subset of T that has a mutation at the specified site, and N is the number of elements of T. T contains all samples included in M and, in addition, those samples that are VAF = 0 at the specified site. Elements of T are denoted as t and m is an element of M. The f_t is a VAF when a sample t is specified, r_m is the total read count when a sample m is specified, and a_m is the mutation read count of the same m specified in r_m.

$$g = \frac{1}{N}\sum_{t\in T} f_t = \frac{1}{N}\sum_{m\in M}\frac{a_m}{r_m} = E_{\text{Mutant}}[\text{VAF}] \tag{1}$$

As the formula indicates, this g is given by a simple mean of VAFs considering

non-mutated samples (VAF = 0). If these values of VAF = 0 are ignored, the value of g will be the mean excess function in EVT calculated with VAF data and a threshold at zero. In the real large-scale genome dataset, a certain threshold is set against multiple values from multiple samples, and a mutation is recorded only if these VAFs exceeded the threshold.

When we consider these critical aspects of VAF in cancer driver mutations, the value of the aggregated tumor VAF (ATVAF) of a cancer driver mutation is equal to the mutant allele frequency among tumors (MFaT) defined as a ratio of mutant samples to total samples in the dataset (see Discussion section).

Demonstration for Fréchet Plot

The cumulative distribution function of GEV $F_{\text{GEV}}(s)$ with three parameters (shape ξ, scale σ, and location μ) is as follows:

$$F_{\text{GEV}}(s) = \exp\left[-A(s)^{-\frac{1}{\xi}}\right] \qquad \left(\xi \neq 0 \;\; ; \;\; A(s) = 1 + \xi\frac{s-\mu}{\sigma} > 0\right) \tag{2}$$

We assume that $A(s)$ is approximately proportional to s (i.e., $A(s) \propto s$). Then,

$$-\ln(-\ln F_{\text{GEV}}(s)) = \frac{1}{\xi}\ln(A(s)) \quad \propto \quad \ln(s) \;\; . \tag{3}$$

Thus, in the Fréchet plot, we have a positive logarithm $\ln(s)$ in the x-axis and a double negative-logarithm $-\ln(-\ln F_{\text{Empirical}}(s))$ in the y-axis assigning observed MFaT to s. Linearity in the Fréchet plot suggests the proportionality ($A(s) \propto s$) and the goodness-of-fit of F_{GEV} to $F_{\text{Empirical}}$.

List of Supplementary Files

S1 Fig. The SSWM population dynamics of cells and the additivity of fitness effects of mutant alleles. (PDF)

S2 Fig. Exploratory plots on cancer driver mutation MFaTs in the total-tumor analysis with position-based filtering. (PDF)

S1 Appendix. Supplementary Materials. (PDF)

S1 Script. get_IcgcProteinAltering.py. (PY)

S1 File. database_ICGC_temp_PostMax.tsv.gz. (GZ)

S2 File. database_COSMIC_temp_PostMax.tsv.gz. (GZ)

S3 File. database_CHANG_temp_PostMax.tsv.gz. (GZ)

S4 File. driver_TotalGene_database_IntOGen.tsv. (TSV)

S5 File. driver_TotalGene_database_CGC.tsv. (TSV)

S6 File. driver_TotalGene_database_Tokheim.tsv. (TSV)

S7 File. driver_TotalSite_database_IntOGen.tsv. (TSV)

S8 File. driver_TotalSite_database_DoCM.tsv. (TSV)

S9 File. driver_TotalSite_database_Bailey.tsv. (TSV)

S10 File. database_RTCGA_temp_Format.tsv.gz. (GZ)

S11 File. database_RTCGA_temp_ProteinAlteringSnv.tsv.gz. (GZ)

S12 File. database_RTCGA_temp_ProteinAlteringSnvDoubleton.tsv.gz. (GZ)

S13 File. database_RTCGA_temp_PostMaxTotal.tsv. (TSV)

S14 File. database_RTCGA_temp_PostMaxType.tsv. (TSV)

S15 File. driver_TypeGene_database_IntOGen.tsv. (TSV)

S16 File. drivertype_RTCGA-IntOGen_temp_PreRank.tsv. (TSV)

S17 File. R_analysis_TotalTumor.zip. (ZIP)

S18 File. R_analysis_TumorTypeSpecific.zip. (ZIP)

S19 File. table_NormalizedEap.tsv. (TSV)

Supporting Information on Google Drive

`https://drive.google.com/file/d/1rzJjD2NsCno0FPgtYsGcoxL-UDPRWG96/view?usp=sharing`

Full Archive on Google Drive

`https://drive.google.com/drive/folders/1EPdSVqlSEXB7_HUison2LLdvo7TL0Ky0?usp=sharing`

Table 1. (Appendix) The list of estimated beneficial mutation effects for BLCA tumor type.

Tumor Type	Mutation	EAP value
BLCA	FGFR3::S249C	0.053844
BLCA	TP53::R248Q	0.04689
BLCA	KDM6A::Q555*	0.040126
BLCA	TP53::E285K	0.027265
BLCA	TP53::R280T	0.021258
BLCA	TP53::E271K	0.021258
BLCA	TP53::Q192*	0.021258
BLCA	ZNF814::D404E	0.021258
BLCA	AHNAK::S4150F	0.016009
BLCA	ERBB3::V104L	0.016009
BLCA	TP53::R273C	0.016009
BLCA	TP53::R175H	0.016009
BLCA	TP53::A159V	0.016009
BLCA	ERCC2::N238S	0.016009
BLCA	NFE2L2::E79K	0.016009
BLCA	NFE2L2::R34G	0.016009
BLCA	SF3B1::E902K	0.016009
BLCA	FGFR3::G380R	0.016009
BLCA	STAG2::Q593*	0.016009

The fields in the Mutation column indicate gene versus amino acid mutation pairs. Beneficial mutation effects are quantified with EAP estimates. Here, EAP stands for expected à posteriori.

Table 2. (Appendix) The list of estimated beneficial mutation effects for BRCA tumor type.

Tumor Type	Mutation	EAP value
BRCA	PIK3CA::H1047R	0.110603
BRCA	PIK3CA::E545K	0.050545
BRCA	PIK3CA::E542K	0.033693
BRCA	TP53::R175H	0.015812
BRCA	TP53::R196*	0.008504
BRCA	PIK3CA::N345K	0.007475
BRCA	TP53::R273H	0.006457
BRCA	TP53::R213*	0.006457
BRCA	TP53::H193R	0.006457
BRCA	TP53::R248W	0.005459
BRCA	TP53::Y220C	0.004492
BRCA	TP53::I195T	0.004492
BRCA	TP53::C176F	0.004492
BRCA	SF3B1::K700E	0.004492
BRCA	CDH1::Q23*	0.003571
BRCA	TP53::R342*	0.003571
BRCA	ERBB2::L755S	0.003571
BRCA	PIK3CA::E726K	0.003571
BRCA	FOXA1::S250F	0.002727
BRCA	CDH1::R335*	0.002727
BRCA	TP53::Q331*	0.002727
BRCA	TP53::R306*	0.002727
BRCA	TP53::E285K	0.002727
BRCA	TP53::R273C	0.002727
BRCA	TP53::G266E	0.002727
BRCA	TP53::G245D	0.002727
BRCA	TP53::H179R	0.002727
BRCA	TP53::C141Y	0.002727
BRCA	PIK3CA::G118D	0.002727
BRCA	PIK3CA::C420R	0.002727
BRCA	PIK3CA::E453K	0.002727
BRCA	PIK3CA::Q546K	0.002727
BRCA	PIK3CA::Q546R	0.002727

The fields in the Mutation column indicate gene versus amino acid mutation pairs. Beneficial mutation effects are quantified with EAP estimates. Here, EAP stands for expected à posteriori.

Table 3. (Appendix) The list of estimated beneficial mutation effects for HNSC tumor type.

Tumor Type	Mutation	EAP value
HNSC	CDKN2A::R80*	0.03806
HNSC	CDKN2A::R58*	0.024643
HNSC	TP53::R282W	0.018413
HNSC	TP53::R175H	0.018413
HNSC	PIK3CA::E542K	0.015465
HNSC	PIK3CA::E545K	0.015465
HNSC	TP53::R273H	0.012629
HNSC	TP53::G245S	0.012629
HNSC	TP53::R213*	0.012629
HNSC	CDKN2A::E120*	0.012629
HNSC	TP53::R306*	0.009932
HNSC	TP53::R248Q	0.009932
HNSC	TP53::H193L	0.009932
HNSC	TP53::H179R	0.009932
HNSC	PIK3CA::H1047R	0.009932
HNSC	CDKN2A::W110*	0.009932
HNSC	HRAS::G13V	0.007561
HNSC	TP53::E298*	0.007561
HNSC	TP53::E285K	0.007561
HNSC	TP53::C275F	0.007561
HNSC	TP53::G266E	0.007561
HNSC	TP53::R248W	0.007561
HNSC	TP53::G245V	0.007561
HNSC	TP53::C242F	0.007561
HNSC	TP53::Y236C	0.007561
HNSC	TP53::Y220C	0.007561
HNSC	TP53::V173M	0.007561
HNSC	TP53::V157F	0.007561
HNSC	TP53::R110L	0.007561
HNSC	NFE2L2::E79Q	0.007561
HNSC	FBXW7::R505G	0.007561
HNSC	CDKN2A::E88*	0.007561
HNSC	ATP6AP2::E119Q	0.007561
HNSC	KDM6A::R519*	0.007561

The fields in the Mutation column indicate gene versus amino acid mutation pairs. Beneficial mutation effects are quantified with EAP estimates. Here, EAP stands for expected à posteriori.

Table 4. (Appendix) The list of estimated beneficial mutation effects for LIHC tumor type.

Tumor Type	Mutation	EAP value
LIHC	TP53::R249S	0.031029
LIHC	CTNNB1::S33P	0.018059
LIHC	CTNNB1::K335I	0.018059
LIHC	TP53::H193R	0.014094
LIHC	CTNNB1::H36P	0.014094
LIHC	CTNNB1::D32G	0.014094
LIHC	TP53::R158H	0.010632
LIHC	TP53::V157F	0.010632
LIHC	IDH1::R132C	0.010632
LIHC	CTNNB1::S45P	0.010632
LIHC	PIK3CA::H1047R	0.010632

The fields in the Mutation column indicate gene versus amino acid mutation pairs. Beneficial mutation effects are quantified with EAP estimates. Here, EAP stands for expected à posteriori.

Table 5. (Appendix) The list of estimated beneficial mutation effects for LUAD tumor type.

Tumor Type	Mutation	EAP value
LUAD	KRAS::G12C	0.114922
LUAD	KRAS::G12V	0.066458
LUAD	U2AF1::S34F	0.023838
LUAD	EGFR::L858R	0.019871
LUAD	BRAF::V600E	0.012344
LUAD	TP53::R337L	0.009169
LUAD	TP53::C277F	0.009169
LUAD	TP53::Q192*	0.009169
LUAD	TP53::A159P	0.009169
LUAD	TP53::R158L	0.009169
LUAD	TP53::R110L	0.009169
LUAD	STK11::W239C	0.009169
LUAD	CTNNB1::S37F	0.009169
LUAD	KDR::A163E	0.009169
LUAD	EGFR::L861Q	0.009169
LUAD	BRAF::G469V	0.009169

The fields in the Mutation column indicate gene versus amino acid mutation pairs. Beneficial mutation effects are quantified with EAP estimates. Here, EAP stands for expected à posteriori.

Table 6. (Appendix) The list of estimated beneficial mutation effects for PRAD tumor type.

Tumor Type	Mutation	EAP value
PRAD	SPOP::F133V	0.01271
PRAD	SPOP::W131G	0.01271
PRAD	HRAS::Q61R	0.007547
PRAD	TP53::C141G	0.007547
PRAD	SPOP::F133C	0.007547
PRAD	SPOP::F102V	0.007547
PRAD	SPOP::Y87N	0.007547
PRAD	MED12::V1223L	0.007547

The fields in the Mutation column indicate gene versus amino acid mutation pairs. Beneficial mutation effects are quantified with EAP estimates. Here, EAP stands for expected à posteriori.

Table 7. (Appendix) The list of estimated beneficial mutation effects for SKCM tumor type.

Tumor Type	Mutation	EAP value
SKCM	BRAF::V600E	0.314887
SKCM	NRAS::Q61R	0.097438
SKCM	NRAS::Q61K	0.073983
SKCM	IDH1::R132C	0.027407
SKCM	MAP2K1::P124S	0.016127
SKCM	CDKN2A::P114L	0.016127
SKCM	PPP6C::R301C	0.013401
SKCM	ARID2::S297F	0.010735
SKCM	NF1::R440*	0.010735
SKCM	SF3B1::R625H	0.010735
SKCM	RAC1::P29S	0.010735
SKCM	CDKN2A::R80*	0.010735
SKCM	TP53::S241F	0.008195
SKCM	TP53::R213*	0.008195
SKCM	TP53::R196*	0.008195
SKCM	SMURF2::R427C	0.008195
SKCM	PCDH18::E569K	0.008195
SKCM	PCDH18::S170F	0.008195
SKCM	BRAF::K601E	0.008195
SKCM	BRAF::G466E	0.008195

The fields in the Mutation column indicate gene versus amino acid mutation pairs. Beneficial mutation effects are quantified with EAP estimates. Here, EAP stands for expected à posteriori.

Table 8. (Appendix) The list of estimated beneficial mutation effects for BLCA tumor type.

Tumor Type	Mutation	EAP value
THCA	BRAF::V600E	0.527742
THCA	NRAS::Q61R	0.072031
THCA	HRAS::Q61R	0.025913
THCA	NRAS::Q61K	0.017904
THCA	HRAS::Q61K	0.007648

The fields in the Mutation column indicate gene versus amino acid mutation pairs. Beneficial mutation effects are quantified with EAP estimates. Here, EAP stands for expected à posteriori.
