# Supplementary material for: Extreme value theory as a framework for understanding mutation frequency distribution in cancer genomes
Source: PLoS One. 2021 Aug 23;16(8):e0243595. doi: 10.1371/journal.pone.0243595 (PMC8382180; doi:10.1371/journal.pone.0243595)
Supplement: Attachment — Submitted filename: rebuttal_Review01_v14.pdf [file pone.0243595.s024.pdf]

First, we wish to thank reviewers for providing critical insights and many good suggestions. We revised our manuscript based on these suggestions. We think our manuscript has been improved in terms of structure, clarity, correctness, and connection to existing research.

The point-to-point response:

Reviewer #1:
Point #1-1: Insufficient framing and connection.
The main issue is the insufficient framing of the study within existing work, beginning with the abstract claiming that "Currently, there is no recognized population genetics framework describing the population dynamics of cancer cells that is applicable to real cancer genome data." The meaning of the last part of the sentence is unclear to me, but in any case, there have been many studies analyzing cancer mutations under population genetics frameworks. For example:

Iwasa, Y., Michor, F., Komarova, N.L. and Nowak, M.A., 2005. Population genetics of tumor suppressor genes. Journal of theoretical biology, 233(1), pp.15-23.

Attolini, C.S.O., Cheng, Y.K., Beroukhim, R., Getz, G., Abdel-Wahab, O., Levine, R.L., Mellinghoff, I.K. and Michor, F., 2010. A mathematical framework to determine the temporal sequence of somatic genetic events in cancer. Proceedings of the National Academy of Sciences, 107(41), pp.17604-17609.

Durrett, R. Population genetics of neutral mutations in exponentially growing cancer cell populations. Ann. Appl. Probabil. 23, 230–250 (2013).

Hu, Z., Sun, R. and Curtis, C., 2017. A population genetics perspective on the determinants of intra-tumor heterogeneity. Biochimica et Biophysica Acta (BBA)-Reviews on Cancer, 1867(2), pp.109-126.

Niida, A., Iwasaki, W.M. and Innan, H., 2018. Neutral theory in cancer cell population genetics. Molecular biology and evolution, 35(6), pp.1316-1321.

Caravagna, G., Heide, T., Williams, M. J., Zapata, L., Nichol, D., Chkhaidze, K., ... & Chesler, L. (2020). Subclonal reconstruction of tumors by using machine learning and population genetics. Nature Genetics, 52(9), 898-907.

The Introduction cites studies in reference to elements of the proposed framework, but omits much research that seems similar to the study as a whole. And in regards to extreme value theory, a paper with important background on EVT applied to beneficial mutations is listed in the references (#41) but doesn't seem to actually be cited in the manuscript.

Without such connections to related existing research, it is unclear whether the framework is novel or replicates existing methods.

Response to the Point #1-1:
We rewrote and re-organized the Introduction section in order to clarify the framing of our work within antecedent studies.

1. More than 50 new reference papers including six papers that the reviewer kindly referred to are cited in the Introduction section.
2. The EVT background paper (formerly reference #41, in this revised manuscript #20, Orr HA, 2010) is cited twice (L29 and L106).
3. In addition, we included the following elements in the Introduction to provide sufficient connection to existing research.
    - An important population genetics model (the mutation-selection balance; MSB) that seems similar to the strong selection and weak mutation (SSWM) model in context of cancer evolution (L23-L44).
    - SSWM model applications to cancer evolution after the clonal expansion (L51-L63).

- Knudson's Two Hit hypothesis (L19-L22).
- Mode of somatic cell division (L139-L151).

Point #1-2: MFaT is doubly described.
MFaT is defined in two subsections ("The Definition and Calculation of MFaT" in Methods and "The Definition of MFaT" in Results). This seems excessive, especially since it gives the impression of a novel metric, even though the fraction of tumors with a particular mutation is a very common statistic in cancer genomics studies.

Response to the Point #1-2:
We removed one MFaT definition from the Results section. The related description is merged to the MFaT definition in the Materials and Methods section (L312-L335). Although the fraction of tumors with a particular mutation is a common statistic, we need to avoid confusion with another common statistic, VAF. To achieve this, explanatory paragraphs are added to the Introduction section (L232-L247).

Point #1-3: Limited description of diploid genetics.
Page 17: "In the field of population genetics, a lot of effort has been devoted solely to monoploid organisms. To translate this knowledge to population genetics of cancer cells, we need an additional assumption for the fitness of heterozygotes: the fitness of a heterozygote is exactly intermediate between the two associated homozygotes." This seems to imply that diploid organisms have been insufficiently studied in population genetics, when in fact, much of population genetics has been devoted to the study of diploid organisms (especially humans), with dominance being a crucial phenomenon in the models. Similarly, the assumption of heterozygote fitness being exactly intermediate is rarely true for cancer mutations.

Response to the Point #1-3:
We clarified the related assumption, approximation and condition. The item "The Additivity Assumption" is renamed to "The Additive Dominance Assumption" to clarify the extent to which the assumption remains approximately valid (L653-L666). Mutations in oncogenes and tumor suppressor genes are treated as exceptions (L660-L666). In addition, the dominance coefficient in relation to cancer biology is discussed in the Introduction section (L29, L42, and L97-L151).

Point #1-4: Lack of justification on no genomic epistasis.
Page 17/18: The framework assumes there is no genomic epistasis, but important cancer mutations often exhibit epistasis, for example between mutations with redundant effects in the same pathway. More justification should be given that this assumption is not overly violated in practice.

Response to the Point #1-4: An item is added.
A new item "The No Epistasis Assumption" is added to the Discussion section (L667-L675). In the item, the complexity of tumor phenotype and its associated pathways are discussed (L670-L675).

Point #1-5: Italicizing gene symbols
Gene names should be italicized.

Response to the Point #1-5:
We italicized gene names.

Point #1-6: Spell missing (caner -> cancer)
Line 614, caner -> cancer

Response to the Point #1-6:
We corrected the misspelling.

Reviewer #2:
Point #2-1: Cartoon of the model.
The manuscript is hard to understand, especially for a more general audience of PLOS ONE, versus a more specialized journal. In particular, the "model" is not very well-described. The data appear to be mutation frequencies, where a limited number of driver mutations (in aggregate) have high mutation frequencies (MFaT) but most driver mutations have very low mutation frequencies. This phenomenon is well-described and well-known. The manuscript could better describe, perhaps with a cartoon exactly what is being modeled biologically. Otherwise, it appears to be mainly a curve-fitting type of exercise, which is interesting but of uncertain significance.

Response to the Point #2-1: A new figure is added.
A new figure with four panels including cartoons is added so as to clarify our model (Figure 1). And this figure was referred to several times in the Introduction (L67, L102-L104, and L130) section. At the same time, we reorganized and rewrote the Introduction section so as to clarify our assumptions for the models as well as the model itself. More than 50 new reference papers cited in the Introduction section in order to clarify the framing of our work within antecedent studies. In addition, we included the following elements in the Introduction to provide sufficient connection to existing research.

- An important population genetics model (the mutation-selection balance; MSB) that seems similar to the strong selection and weak mutation (SSWM) model in context of cancer evolution (L23-L44).
- SSWM model applications to cancer evolution after the clonal expansion (L51-L63).
- Knudson's Two Hit hypothesis (L19-L22).
- Mode of somatic cell division (L139-L151).

Point #2-2: Lack of reference to adjustment of sample purity.
The data do not adjust for sample purity, which will reinforce the low frequency driver mutations

Response to the Point #2-2: Reference added.
As the reviewer implied, large-scale genomics datasets have different levels of sample purity. Therefore, we restricted ourselves to separated analysis within each dataset. Adjusting datasets each containing tens of thousands of specimens for sample purity is often computationally impractical, leading to a shared agreement that referring to the database name in combination with an original filtering method is sufficient. For example:

Wong, Wing Chung, et al. "CHASM and SNVBox: toolkit for detecting biologically important single nucleotide mutations in cancer." Bioinformatics 27.15 (2011): 2147-2148.

Mao, Yong, et al. "CanDrA: cancer-specific driver missense mutation annotation with optimized features." PloS one 8.10 (2013): e77945.

Cisneros, Luis, et al. "Ancient genes establish stress-induced mutation as a hallmark of cancer." PLoS One 12.4 (2017): e0176258.

However, to ensure consistency of the sample purity, it is widely accepted to refer to the original paper of such a dataset in which mutation filtering criteria are described. Thus, we referred to such literatures in the "Data Processing" item in the Materials and Methods section (L294-L296).

Point #2-3: Unclear graph representation.
It seems unlikely that "all" cancers follow the same rules or even have the same driver mutations. Yet the manuscript combines multiple types of cancer. It is uncertain if the analysis works better for individual cancer type, or if the analysis works better when combining all the data (ie as in Figure 1). It would be helpful if some of the peaks with high MFat (TP53?) could be identified in Fig1 A.

Response to the Point #2-3:

With sincere respect to the points that the reviewer made, we improved appearance of the graphs. In all density plots of total-tumor MFaTs, mutations of the highest MFaTs are indicated (Figs 2A, 3A, and 4A).

Point #2-4: Lack of clarity and troublesome descriptions.
Along these lines, it is unclear if different mutations in the same gene but at different nucleotides are counted as a “driver” mutation. (Does TP53 count as a single driver or can it be different drivers?---this reviewer has trouble with the description on page 4)

Response to the Point #2-4:
To address this issue, we replaced old text and added a new item. The original contents of the item "Data Processing" is now moved to S1 Appendix and newer text is supplied in the item (L293-L310). In addition, a new item "Study Design" is added to clarify the method of counting driver mutations (L249-L262).

Point #2-5: Low frequency potential driver mutations.
It would be interesting or perhaps informative if the authors also looked at passenger mutations (such as TTN) to check if the distributions do or do not follow an EVT type of distribution. This is optional, but could be interesting because of the uncertainty of whether many low frequency “driver” mutations are in fact driver mutations.

Response to the Point #2-5:
The suggestion is remarkably interesting. But, in current manuscript, we wish to concentrate on establishing and presenting the EVT framework. Also, we feel one needs some careful considerations involving effect sizes of mutations (Some of our assumptions hold exclusively for driver genes). We hope to pursue this passenger mutation problem as a separate study in future. Thank you very much for your great suggestion.

Point #2-6: The table of calculated fitness effects.
The paper concludes that EVT and the analysis can help estimate the fitness of beneficial mutations. It would be useful if the authors provide a list of beneficial mutations and their estimated fitness from their analysis (if possible).

Response to the Point #2-6:
We added tables of estimated driver fitness effects for each cancer types in S1 Appendix (Appendix Tables 1-8). In addition, the single table for these tumor types is included in the Supplementary Files (S19 File table NormalizedEap.tsv). We hope these tables will be useful.

EOF
