# Supplementary material for: Extreme value theory as a framework for understanding mutation frequency distribution in cancer genomes
Source: PLoS One. 2021 Aug 23;16(8):e0243595. doi: 10.1371/journal.pone.0243595 (PMC8382180; doi:10.1371/journal.pone.0243595)
Supplement: Attachment — Submitted filename: Response to Reviewers_v03.pdf [file pone.0243595.s025.pdf]

Reviewer #1: (No Response)

Reviewer #2: The manuscript continues to be very difficult to understand. The concepts and the logic seem insufficient for a more general readership that is seen with PLOS ONE. The added "cartoon" (Fig 1) illustrates general principles, but does not seem to allow a reader to "see" how extreme value theory is being applied in the paper.

We completely changed Fig 1 to clarify not only the medical and cell-biological aspects of our study, but also the point where extreme value theory (EVT) is applied to the cellular fitness landscape (Fig 1A, B, D). We also made big modification in the Introduction part. In the first section (The “Big Bang” Model of Cancer Development and Population Genetics of Cancer Cells) in Introduction, the concepts and the logic are made more straightforward for the reader's ease of understanding. We wish these changes, in combination with the schematic S1FigC in Supporting information, may allow a reader to "see" the way by which EVT is applied in the paper.

Reviewer #3: This is an interesting paper that applies the extreme value theory to model the mutation frequency distribution in the cancer genome. The authors showed that the driver mutation count distribution can be well fitted by the Frechet-type extreme distribution across varying cancer genomic datasets (e.g. TCGA, ICGC, etc). Based on the model fitting, the authors concluded that early tumor evolution (pre-clonal expansion) likely follows the classic strong selection and weak mutation (SSWM) regime in population genetics theory.

1) It’s not clear to me regarding the relationship between SSWM model and the extreme value distribution. Can the authors provide a mathematical prove that the SSWM regime of tumor evolution will give rise to Frechet-type extreme value distribution for driver mutation number? Or others have proven this?

There is no direct relationship between the SSWM model and the extreme value distribution. However, the SSWM model is important here as a prerequisite for an assumption that the adaptation process is Markov process. This allows us to assume that only mutations with fitness effects which is the maximum or above a certain threshold will fix in each adaptation step. The "above the threshold" model gives rise to Generalized Pareto Distribution (GPD); and the "maxima" model gives rise to Generalized Extreme Value distribution (GEV).

More precisely, Joyce et al (Joyce et al, 2008) demonstrated that the fitness landscape of adaptation of DNA sequence would follow GPD under the assumption of the SSWM and the "above the threshold" model. In this paper, we used the "block maxima" model because it fitted better with the "Big Bang" model of the cancer evolution. The Fisher-Tippett-Gnedenko theorem proves that the maxima from independent and identically distributed random variables will follow GEV distributions.

(Joyce P et al, 2008): Joyce, Paul, et al. "A general extreme value theory model for the adaptation of DNA sequences under strong selection and weak mutation." Genetics 180.3 (2008): 1627-1643.

2) I don’t understand why the authors focused on specific site of driver genes? It seems gene-level mutation distribution fits the Frechet-type distribution better than site-level distribution (Fig 2 and 3). I suggest the authors to show gene-level mutation distribution

fitting in main figures and site-level mutation distribution in supplementary data. Also, Fig 2a and 3a are both site-level fitting, why it was said Fig 2 is on gene level while Fig 3 is site level.

Classical population genetics theories analyze evolution of a DNA sequence as a set of nucleotide residues instead of gene symbols (e.g., (Orr HA, 2002)). To perform "gene-level" analysis, we imagine that more careful formulation and study design are necessary. Thus, in fact, all the analyses performed in this paper were at "site-level". We wish that we have a chance to design "gene-level" analysis in a separate study in future.

We agree that the formerly Fig 3A (now S2FigA) shows lack of data points in ICGC cases. We have moved the formerly Fig 3 figures to Supporting information. Also, misleading labels in the figure and table texts (i.e., "driver-gene definition" and "driver-site definition") are now replaced with clearer ones (i.e., "symbol-based filtering" and "position-based filtering"). In addition, the corresponding descriptions in Results (L466-L467, L486-L487, L496) and Discussion (L617) are updated.

(Orr HA, 2002): Orr, H. Allen. "The population genetics of adaptation: the adaptation of DNA sequences." Evolution 56.7 (2002): 1317-1330.

3) Is the dominance h (Fig. 1D) important in the mathematical analysis? I didn’t see any information regarding h in results.

We agree that although the dominance coefficient h may serve our understanding, it is less important in the context of the Introduction part. The details of this coefficient are omitted from Introduction and its related figures are moved to Supporting information (S1FigA, S1FigB).

4) The introduction section is too long and should be cutted and many of the information is misleading.

We removed redundancy in the description and made it more straightforward, especially in the first section of the introduction. As a result, the introduction part has decreased by 24 % in word count (3088 -> 2354 words, citations and formula included). We wish the description in the section has become more concise, straightforward, and clear.

(The reviewers’ comments were shown in blue letters and our response in black.)
